# 2-[(5-Chloro-2-oxidobenzyl­idene)aza­nium­yl]-2-methyl­propane-1,3-diol

**DOI:** 10.1107/S1600536811055784

**Published:** 2012-01-07

**Authors:** Dong-Yue Wang, Min Liu, Jing-Jun Ma

**Affiliations:** a Hebei Key Laboratory of Bioinorganic Chemistry, College of Sciences, Agricultural University of Hebei, Baoding 071001, People’s Republic of China

## Abstract

The title compound, C_11_H_14_ClNO_3_, was prepared by the condensation of equimolar quanti­ties of 5-chloro­salicyl­aldehyde and 2-amino-2-methyl­propane-1,3-diol in methanol. In the crystal, it exists in the zwitterionic form, with nominal proton transfer from the phenol group to the imine N atom. This results in the formation of an intra­molecular N—H⋯O hydrogen bond, which generates an *S*(6) ring. Inter­molecular O—H⋯O hydrogen bonds arise from the hy­droxy groups, forming (001) sheets.

## Related literature

For a related structure we have reported recently and for background to Schiff bases, see: Wang *et al.* (2011 ▶). For standard bond lengths, see: Allen *et al.* (1987 ▶).
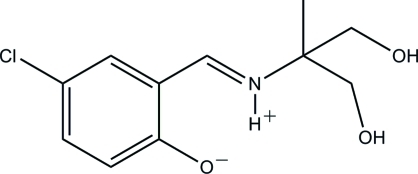


## Experimental

### 

#### Crystal data

C_11_H_14_ClNO_3_
*M* *_r_* = 243.68
Orthorhombic, 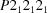

*a* = 6.0019 (16) Å
*b* = 8.838 (3) Å
*c* = 21.555 (3) Å
*V* = 1143.4 (5) Å^3^
*Z* = 4
Mo *K*α radiation
μ = 0.33 mm^−1^
*T* = 298 K
0.13 × 0.12 × 0.10 mm

#### Data collection

Bruker SMART 1K CCD diffractometer
Absorption correction: multi-scan (*SADABS*; Sheldrick, 1996 ▶) *T*_min_ = 0.959, *T*_max_ = 0.968
5744 measured reflections
2105 independent reflections
1745 reflections with *I* > 2σ(*I*)
*R*_int_ = 0.033

#### Refinement

*R*[*F* ^2^ > 2σ(*F* ^2^)] = 0.039
*wR*(*F* ^2^) = 0.076
*S* = 1.05
2105 reflections
155 parameters
3 restraints
H atoms treated by a mixture of independent and constrained refinement
Δρ_max_ = 0.17 e Å^−3^
Δρ_min_ = −0.15 e Å^−3^
Absolute structure: Flack (1983 ▶), 842 Friedel pairs
Flack parameter: −0.06 (8)

### 

Data collection: *SMART* (Bruker, 2007 ▶); cell refinement: *SAINT* (Bruker, 2007 ▶); data reduction: *SAINT*; program(s) used to solve structure: *SHELXS97* (Sheldrick, 2008 ▶); program(s) used to refine structure: *SHELXL97* (Sheldrick, 2008 ▶); molecular graphics: *SHELXTL* (Sheldrick, 2008 ▶); software used to prepare material for publication: *SHELXTL*.

## Supplementary Material

Crystal structure: contains datablock(s) I, global. DOI: 10.1107/S1600536811055784/hb6578sup1.cif

Structure factors: contains datablock(s) I. DOI: 10.1107/S1600536811055784/hb6578Isup2.hkl

Supplementary material file. DOI: 10.1107/S1600536811055784/hb6578Isup3.cml

Additional supplementary materials:  crystallographic information; 3D view; checkCIF report

## Figures and Tables

**Table 1:** Hydrogen-bond geometry (Å, °)

*D*—H⋯*A*	*D*—H	H⋯*A*	*D*⋯*A*	*D*—H⋯*A*
N1—H1⋯O1	0.90 (1)	1.84 (2)	2.606 (3)	142 (2)
O4—H4⋯O3^i^	0.85 (1)	1.87 (1)	2.680 (2)	160 (3)
O3—H3*A*⋯O1^ii^	0.85 (1)	1.80 (1)	2.648 (2)	176 (3)

Symmetry codes: (i) 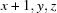; (ii) 
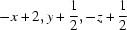
.

**Fig. 1.**
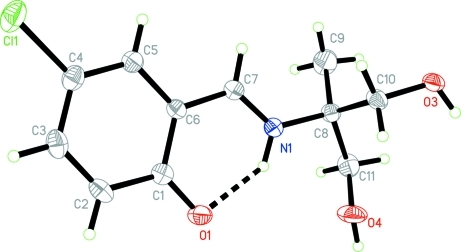
The molecular structure of the title compound, with displacement ellipsoids drawn at the 30% probability level. Intramolecular N—H···O hydrogen bond is drawn as a dashed line.

**Fig. 2.**
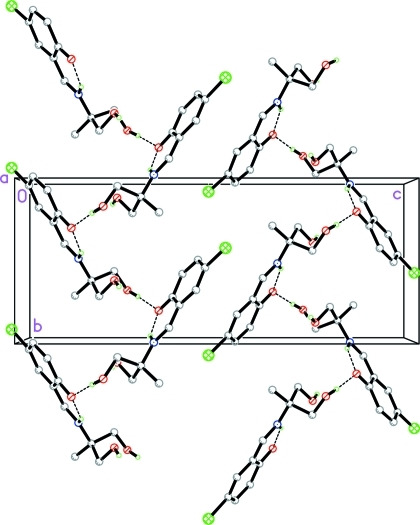
Fragments of (001) sheets moleucles of the title compound, viewed along the a axis.

## supplementary crystallographic information

### Comment

Recently, we have reported the structures of a few Schiff base compounds (e.g. Wang *et al.*, 2011). As a continuation of the work, we present here the crystal structure of the title compound, that was obtained as the product of the reaction of 5-chlorosalicylaldehyde with 2-amino-2-methylpropane-1,3-diol in methanol.

In the title compound, Fig. 1, there in an intramolecular N1—H1···O1 hydrogen bond (Table 1). The bond distances and angles are within normal ranges (Allen *et al.*, 1987).

In the crystal of the compound, the Schiff base molecules are linked through intermolecular O—H···O hydrogen bond, to form (001) sheets. (Table 1 and Fig. 2).

### Experimental

To a methanol solution (10 ml) of 5-chlorosalicylaldehyde (0.1 mmol, 15.6 mg) and 2-amino-2-methylpropane-1,3-diol (0.1 mmol, 10.5 mg), a few drops of acetic acid were added. The mixture was refluxed for 1 h and then cooled to room temperature. The yellow crystalline solid was collected by filtration, washed with cold methanol and dried in air. Yellow blocks were obtained by slow evaporation of a methanol solution of the product in air.

### Refinement

The NH and OH H-atoms were located in a difference Fourier map and were refined with distance restraints, N—H = 0.90 (1) Å, and O—H = 0.85 (1) Å. The C-bound H atoms were positioned geometrically and refined using a riding model, with C—H = 0.93–0.97 Å, with *U*_iso_(H) = 1.2*U*_eq_(C).

### Crystal data

**Table:**

C_11_H_14_ClNO_3_	*D*_x_ = 1.416 Mg m^−^^3^
*M**_r_* = 243.68	Mo *K*α radiation, λ = 0.71073 Å
Orthorhombic, *P*2_1_2_1_2_1_	Cell parameters from 1733 reflections
*a* = 6.0019 (16) Å	θ = 2.5–24.2°
*b* = 8.838 (3) Å	µ = 0.33 mm^−^^1^
*c* = 21.555 (3) Å	*T* = 298 K
*V* = 1143.4 (5) Å^3^	Block, yellow
*Z* = 4	0.13 × 0.12 × 0.10 mm
*F*(000) = 512	

### Data collection

**Table:**

Bruker SMART 1K CCD diffractometer	2105 independent reflections
Radiation source: fine-focus sealed tube	1745 reflections with *I* > 2σ(*I*)
graphite	*R*_int_ = 0.033
ω scan	θ_max_ = 25.5°, θ_min_ = 2.5°
Absorption correction: multi-scan (*SADABS*; Sheldrick, 1996)	*h* = −5→7
*T*_min_ = 0.959, *T*_max_ = 0.968	*k* = −10→10
5744 measured reflections	*l* = −26→21

### Refinement

**Table:**

Refinement on *F*^2^	Secondary atom site location: difference Fourier map
Least-squares matrix: full	Hydrogen site location: inferred from neighbouring sites
*R*[*F*^2^ > 2σ(*F*^2^)] = 0.039	H atoms treated by a mixture of independent and constrained refinement
*wR*(*F*^2^) = 0.076	*w* = 1/[σ^2^(*F*_o_^2^) + (0.0287*P*)^2^ + 0.113*P*] where *P* = (*F*_o_^2^ + 2*F*_c_^2^)/3
*S* = 1.05	(Δ/σ)_max_ < 0.001
2105 reflections	Δρ_max_ = 0.17 e Å^−^^3^
155 parameters	Δρ_min_ = −0.15 e Å^−^^3^
3 restraints	Absolute structure: Flack (1983), 842 Friedel pairs
Primary atom site location: structure-invariant direct methods	Flack parameter: −0.06 (8)

### Special details

**Table:**

Geometry. All e.s.d.'s (except the e.s.d. in the dihedral angle between two l.s. planes) are estimated using the full covariance matrix. The cell e.s.d.'s are taken into account individually in the estimation of e.s.d.'s in distances, angles and torsion angles; correlations between e.s.d.'s in cell parameters are only used when they are defined by crystal symmetry. An approximate (isotropic) treatment of cell e.s.d.'s is used for estimating e.s.d.'s involving l.s. planes.
Refinement. Refinement of *F*^2^ against ALL reflections. The weighted *R*-factor *wR* and goodness of fit *S* are based on *F*^2^, conventional *R*-factors *R* are based on *F*, with *F* set to zero for negative *F*^2^. The threshold expression of *F*^2^ > σ(*F*^2^) is used only for calculating *R*-factors(gt) *etc*. and is not relevant to the choice of reflections for refinement. *R*-factors based on *F*^2^ are statistically about twice as large as those based on *F*, and *R*- factors based on ALL data will be even larger.

### Fractional atomic coordinates and isotropic or equivalent isotropic displacement parameters (Å^2^)

**Table:**

	*x*	*y*	*z*	*U*_iso_*/*U*_eq_	
Cl1	0.67835 (15)	−0.07637 (8)	−0.02077 (3)	0.0613 (2)	
N1	0.8677 (3)	0.4851 (2)	0.15414 (9)	0.0356 (5)	
O1	1.2095 (3)	0.30421 (19)	0.14623 (7)	0.0422 (4)	
O3	0.5214 (3)	0.6832 (2)	0.27003 (8)	0.0462 (5)	
O4	1.0926 (3)	0.6167 (2)	0.24842 (11)	0.0609 (6)	
C1	1.0897 (4)	0.2197 (3)	0.11037 (11)	0.0350 (6)	
C2	1.1701 (5)	0.0789 (3)	0.08645 (11)	0.0442 (6)	
H2	1.3107	0.0449	0.0981	0.053*	
C3	1.0452 (5)	−0.0064 (3)	0.04693 (12)	0.0470 (7)	
H3	1.1032	−0.0965	0.0316	0.056*	
C4	0.8320 (5)	0.0388 (3)	0.02898 (11)	0.0412 (6)	
C5	0.7447 (4)	0.1709 (3)	0.05062 (10)	0.0366 (6)	
H5	0.6031	0.2012	0.0383	0.044*	
C6	0.8698 (4)	0.2620 (3)	0.09192 (10)	0.0315 (5)	
C7	0.7671 (4)	0.3945 (3)	0.11616 (10)	0.0352 (6)	
H7	0.6219	0.4170	0.1042	0.042*	
C8	0.7731 (4)	0.6211 (3)	0.18423 (10)	0.0350 (6)	
C9	0.6345 (6)	0.7147 (3)	0.13884 (12)	0.0603 (8)	
H9A	0.7202	0.7341	0.1021	0.090*	
H9B	0.5940	0.8089	0.1579	0.090*	
H9C	0.5020	0.6598	0.1280	0.090*	
C10	0.6293 (4)	0.5634 (3)	0.23840 (11)	0.0384 (6)	
H10A	0.5181	0.4938	0.2226	0.046*	
H10B	0.7229	0.5085	0.2674	0.046*	
C11	0.9699 (4)	0.7115 (3)	0.20833 (12)	0.0462 (7)	
H11A	1.0627	0.7446	0.1741	0.055*	
H11B	0.9180	0.8002	0.2306	0.055*	
H3A	0.603 (4)	0.720 (3)	0.2983 (10)	0.069*	
H4	1.215 (3)	0.651 (3)	0.2621 (12)	0.069*	
H1	1.005 (2)	0.452 (3)	0.1646 (11)	0.055*	

### Atomic displacement parameters (Å^2^)

**Table:**

	*U*^11^	*U*^22^	*U*^33^	*U*^12^	*U*^13^	*U*^23^
Cl1	0.0825 (6)	0.0435 (4)	0.0579 (4)	−0.0035 (4)	−0.0139 (4)	−0.0124 (3)
N1	0.0328 (13)	0.0364 (11)	0.0375 (11)	0.0047 (10)	−0.0019 (11)	−0.0049 (9)
O1	0.0355 (11)	0.0420 (10)	0.0492 (10)	0.0030 (8)	−0.0089 (9)	0.0012 (8)
O3	0.0260 (10)	0.0535 (11)	0.0593 (13)	0.0027 (9)	−0.0050 (9)	−0.0220 (9)
O4	0.0310 (11)	0.0618 (13)	0.0897 (15)	−0.0013 (10)	−0.0201 (11)	−0.0092 (12)
C1	0.0362 (16)	0.0340 (13)	0.0349 (13)	−0.0015 (11)	0.0023 (11)	0.0063 (11)
C2	0.0376 (15)	0.0406 (14)	0.0543 (15)	0.0119 (14)	0.0050 (14)	0.0048 (13)
C3	0.0590 (19)	0.0330 (13)	0.0491 (16)	0.0070 (14)	0.0076 (15)	−0.0023 (13)
C4	0.0514 (17)	0.0346 (13)	0.0376 (13)	−0.0026 (13)	−0.0027 (13)	−0.0006 (11)
C5	0.0362 (16)	0.0358 (13)	0.0377 (13)	−0.0003 (11)	0.0001 (12)	0.0000 (11)
C6	0.0285 (14)	0.0331 (12)	0.0329 (12)	0.0010 (11)	0.0026 (11)	0.0009 (11)
C7	0.0318 (14)	0.0378 (13)	0.0360 (13)	0.0002 (11)	0.0009 (11)	−0.0011 (11)
C8	0.0351 (15)	0.0286 (12)	0.0412 (13)	0.0039 (11)	−0.0039 (12)	−0.0041 (10)
C9	0.076 (2)	0.0487 (16)	0.0564 (17)	0.0194 (16)	−0.0117 (16)	0.0018 (14)
C10	0.0296 (14)	0.0332 (12)	0.0524 (15)	−0.0012 (12)	0.0023 (12)	−0.0090 (12)
C11	0.0412 (17)	0.0391 (14)	0.0585 (17)	−0.0131 (13)	0.0074 (15)	−0.0071 (13)

### Geometric parameters (Å, °)

**Table:**

Cl1—C4	1.743 (2)	C4—C5	1.362 (3)
N1—C7	1.294 (3)	C5—C6	1.416 (3)
N1—C8	1.479 (3)	C5—H5	0.9300
N1—H1	0.903 (10)	C6—C7	1.423 (3)
O1—C1	1.293 (3)	C7—H7	0.9300
O3—C10	1.416 (3)	C8—C11	1.517 (3)
O3—H3A	0.847 (10)	C8—C9	1.528 (3)
O4—C11	1.411 (3)	C8—C10	1.539 (3)
O4—H4	0.847 (10)	C9—H9A	0.9600
C1—C6	1.428 (3)	C9—H9B	0.9600
C1—C2	1.431 (3)	C9—H9C	0.9600
C2—C3	1.362 (3)	C10—H10A	0.9700
C2—H2	0.9300	C10—H10B	0.9700
C3—C4	1.395 (4)	C11—H11A	0.9700
C3—H3	0.9300	C11—H11B	0.9700
			
C7—N1—C8	126.9 (2)	C6—C7—H7	118.7
C7—N1—H1	112.7 (17)	N1—C8—C11	106.2 (2)
C8—N1—H1	120.1 (17)	N1—C8—C9	111.61 (19)
C10—O3—H3A	112 (2)	C11—C8—C9	111.0 (2)
C11—O4—H4	117 (2)	N1—C8—C10	106.20 (18)
O1—C1—C6	121.9 (2)	C11—C8—C10	110.55 (19)
O1—C1—C2	122.0 (2)	C9—C8—C10	111.1 (2)
C6—C1—C2	116.1 (2)	C8—C9—H9A	109.5
C3—C2—C1	121.4 (2)	C8—C9—H9B	109.5
C3—C2—H2	119.3	H9A—C9—H9B	109.5
C1—C2—H2	119.3	C8—C9—H9C	109.5
C2—C3—C4	121.3 (2)	H9A—C9—H9C	109.5
C2—C3—H3	119.3	H9B—C9—H9C	109.5
C4—C3—H3	119.3	O3—C10—C8	111.98 (19)
C5—C4—C3	120.2 (2)	O3—C10—H10A	109.2
C5—C4—Cl1	120.5 (2)	C8—C10—H10A	109.2
C3—C4—Cl1	119.27 (19)	O3—C10—H10B	109.2
C4—C5—C6	119.9 (2)	C8—C10—H10B	109.2
C4—C5—H5	120.0	H10A—C10—H10B	107.9
C6—C5—H5	120.0	O4—C11—C8	107.65 (19)
C5—C6—C7	118.0 (2)	O4—C11—H11A	110.2
C5—C6—C1	121.1 (2)	C8—C11—H11A	110.2
C7—C6—C1	120.9 (2)	O4—C11—H11B	110.2
N1—C7—C6	122.6 (2)	C8—C11—H11B	110.2
N1—C7—H7	118.7	H11A—C11—H11B	108.5

### Hydrogen-bond geometry (Å, °)

**Table:**

*D*—H···*A*	*D*—H	H···*A*	*D*···*A*	*D*—H···*A*
N1—H1···O1	0.90 (1)	1.84 (2)	2.606 (3)	142 (2)
O4—H4···O3^i^	0.85 (1)	1.87 (1)	2.680 (2)	160 (3)
O3—H3A···O1^ii^	0.85 (1)	1.80 (1)	2.648 (2)	176 (3)

Symmetry codes: (i) *x*+1, *y*, *z*; (ii) −*x*+2, *y*+1/2, −*z*+1/2.
